# Models of Gender Dysphoria Using Social Media Data for Use in Technology-Delivered Interventions: Machine Learning and Natural Language Processing Validation Study

**DOI:** 10.2196/47256

**Published:** 2023-06-16

**Authors:** Cory J Cascalheira, Ryan E Flinn, Yuxuan Zhao, Dannie Klooster, Danica Laprade, Shah Muhammad Hamdi, Jillian R Scheer, Alejandra Gonzalez, Emily M Lund, Ivan N Gomez, Koustuv Saha, Munmun De Choudhury

**Affiliations:** 1 Department of Counseling & Educational Psychology New Mexico State University Las Cruces, NM United States; 2 Department of Psychology Syracuse University Syracuse, NY United States; 3 Augusta University Augusta, GA United States; 4 University of North Dakota Grand Forks, ND United States; 5 Oklahoma State University Stillwater, OK United States; 6 Northern Arizona University Flagstaff, AZ United States; 7 Department of Computer Science Utah State University Logan, UT United States; 8 Xavier University Cincinnati, OH United States; 9 University of Alabama Tuscaloosa, AL United States; 10 Ewha Women’s University Seoul Republic of Korea; 11 University of Illinois at Urbana-Champaign Champaign, IL United States; 12 Georgia Institute of Technology Atlanta, GA United States

**Keywords:** gender diverse, gender dysphoria, social media, social computing, digital health, mobile phone

## Abstract

**Background:**

The optimal treatment for gender dysphoria is medical intervention, but many transgender and nonbinary people face significant treatment barriers when seeking help for gender dysphoria. When untreated, gender dysphoria is associated with depression, anxiety, suicidality, and substance misuse. Technology-delivered interventions for transgender and nonbinary people can be used discretely, safely, and flexibly, thereby reducing treatment barriers and increasing access to psychological interventions to manage distress that accompanies gender dysphoria. Technology-delivered interventions are beginning to incorporate machine learning (ML) and natural language processing (NLP) to automate intervention components and tailor intervention content. A critical step in using ML and NLP in technology-delivered interventions is demonstrating how accurately these methods model clinical constructs.

**Objective:**

This study aimed to determine the preliminary effectiveness of modeling gender dysphoria with ML and NLP, using transgender and nonbinary people’s social media data.

**Methods:**

Overall, 6 ML models and 949 NLP-generated independent variables were used to model gender dysphoria from the text data of 1573 Reddit (Reddit Inc) posts created on transgender- and nonbinary-specific web-based forums. After developing a codebook grounded in clinical science, a research team of clinicians and students experienced in working with transgender and nonbinary clients used qualitative content analysis to determine whether gender dysphoria was present in each Reddit post (ie, the dependent variable). NLP (eg, n-grams, Linguistic Inquiry and Word Count, word embedding, sentiment, and transfer learning) was used to transform the linguistic content of each post into predictors for ML algorithms. A k-fold cross-validation was performed. Hyperparameters were tuned with random search. Feature selection was performed to demonstrate the relative importance of each NLP-generated independent variable in predicting gender dysphoria. Misclassified posts were analyzed to improve future modeling of gender dysphoria.

**Results:**

Results indicated that a supervised ML algorithm (ie, optimized extreme gradient boosting [XGBoost]) modeled gender dysphoria with a high degree of accuracy (0.84), precision (0.83), and speed (1.23 seconds). Of the NLP-generated independent variables, *Diagnostic and Statistical Manual of Mental Disorders, Fifth Edition* (*DSM-5*) clinical keywords (eg, *dysphoria* and *disorder*) were most predictive of gender dysphoria. Misclassifications of gender dysphoria were common in posts that expressed uncertainty, featured a stressful experience unrelated to gender dysphoria, were incorrectly coded, expressed insufficient linguistic markers of gender dysphoria, described past experiences of gender dysphoria, showed evidence of identity exploration, expressed aspects of human sexuality unrelated to gender dysphoria, described socially based gender dysphoria, expressed strong affective or cognitive reactions unrelated to gender dysphoria, or discussed body image.

**Conclusions:**

Findings suggest that ML- and NLP-based models of gender dysphoria have significant potential to be integrated into technology-delivered interventions. The results contribute to the growing evidence on the importance of incorporating ML and NLP designs in clinical science, especially when studying marginalized populations.

## Introduction

### The Impact and Standard Treatment of Gender Dysphoria

Up to 78% of transgender and nonbinary (TNB) people (ie, people whose gender identity or expression differs from the societal expectations of their sex assigned at birth) in the United States report experiencing gender dysphoria before the age of 7 years [1]. *Gender dysphoria* is “the distress that may accompany the incongruence between one’s experienced or expressed gender and one’s assigned gender” [2]. Gender dysphoria can be considered either a medical diagnosis or a symptom [3]. It is often exacerbated by anti-TNB social (eg, stigma, prejudice, discrimination, and violence [4]) and structural (eg, binary concepts of gender [5]) forces. As a construct, gender dysphoria plays a role in the lives of many TNB people. For instance, some TNB people attempting to access health care are diagnosed with gender dysphoria to provide them with access to gender-affirming treatment (eg, hormone therapy and gender affirmation surgeries [6]), even if their dysphoria is minimal; some researchers have identified this practice as a form of health care gatekeeping [3,6,7] because experiencing, or at least reporting, gender dysphoria is often a prerequisite for TNB people to access gender-affirming care. Many TNB people who experience gender dysphoria but receive no treatment experience alarming rates of anxiety, depression, suicidality, and substance misuse [8-10]. As the treatment of gender dysphoria has been shown to reduce negative health outcomes in TNB individuals [9], coping with and minimizing gender dysphoria that causes significant distress is often a treatment goal in both medical and psychological interventions for TNB clients [9].

Existing medical and psychological interventions can be effective at treating and managing gender dysphoria. Gender-affirming medical interventions, such as hormone replacement therapy, gender affirmation surgery, and laser hair removal, are the most effective treatments for gender dysphoria [9,11-13]. Psychological interventions are also valuable [14] but are less effective than medical interventions and primarily help TNB people cope with and manage the distress caused by gender dysphoria.

### Treatment Barriers and the Need for Technology-Delivered Interventions

Unfortunately, TNB people face significant barriers to accessing health care [15,16], which results in inadequate medical treatment for gender dysphoria. Treatment barriers limiting TNB people’s access to clinical care are multifaceted and systemic, including discrimination in the health care system, providers with inadequate knowledge about the health of TNB people, a lack of TNB-specific services, fear of familial rejection, and insufficient coordinated care efforts [15-17]. TNB people are also less likely than cisgender sexual minority people and heterosexual people to access health care [18]. Further, approximately 25% of TNB people report health care avoidance due to anticipation of gender-based discrimination [18], and up to 46% of TNB youths intentionally avoid disclosure of their TNB identity to health care providers [19].

Although these findings indicate a need for more accessible medical treatment options for gender dysphoria, they also suggest that psychological interventions, which can be delivered through technology, can assist in circumventing treatment barriers. Although such technology-delivered interventions should not be a replacement for medical interventions and certainly do not supplant the need for advocacy and policy reform to reduce treatment access barriers for TNB people, they may be an important step toward reducing gender dysphoria in TNB people living in high-stigma and low-resource areas, as these are often places where barriers to receiving gender dysphoria treatment are particularly pronounced [15,17,20]. In fact, the recognition of these treatment barriers has generated increased interest in technology-delivered interventions for promoting health of TNB people (eg, supportive SMS text messages that deliver TNB-affirming cognitive behavioral therapy [21]) because technology-delivered interventions can be used discretely, safely, and flexibly, overcoming many of the treatment barriers TNB people face [22]. However, most existing technology-delivered interventions developed for TNB populations target HIV risk [22]. Only 3 extant technology-delivered interventions target gender-affirming care [22], and none target gender dysphoria specifically.

### Potential Utility of Machine Learning and Natural Language Processing in Gender Dysphoria Treatment

Of the technology-delivered interventions currently available for TNB people [22], none leverage machine learning (ML; ie, the field of study that gives computers the ability to learn without being explicitly programmed) and natural language processing (NLP; ie, computer algorithms that understand patterns in human language) despite the use of these cutting-edge computational methods in technology-delivered interventions for other marginalized populations (eg, men who have sex with men [23]). A high-level introduction to ML and NLP is provided in the study by Goldberg et al [24].

Before ML and NLP can be incorporated into intervention development to enhance intervention delivery (eg, tailoring intervention content to the unique ways in which an individual communicates on social media), researchers must show that ML and NLP can adequately model the clinical construct of interest [23]. Thus, this study sought to use ML, NLP, and the social media content from a nonclinical sample of TNB people to investigate the preliminary effectiveness of using computational methods to model gender dysphoria.

There are several reasons why ML, NLP, and social media warrant examination as potential methods to enhance technology-delivered interventions for gender dysphoria. First, traditional means of measuring mental health symptoms and disorders are limited to self-report, infrequent clinical visits, or inaccessible patient care [25]; the latter 2 are especially significant given the health care access barriers TNB people face [15,16]. By contrast, measuring mental health symptoms using ML and NLP leverages the popularity and accessibility of social media, as well as the self-disclosure encouraged by these platforms [26], to detect and model clinical symptoms and disorders accurately [27,28].

Second, technology-delivered interventions tend to be feasible and acceptable among TNB people, with TNB people generally adhering to these interventions and perceiving them as personalized and convenient [22]. If ML and NLP can model gender dysphoria and the results can be incorporated into downstream technology-delivered interventions to further individualize content, then this computational approach may be well received by TNB people.

Third, TNB people use social media to elicit or provide TNB-specific social support (eg, validation of TNB identity and suggestions on how to obtain gender-affirming treatment [29]). Hence, TNB people with inadequate health care access may seek peer assistance in coping with gender dysphoria on social media. By seeking peer assistance on social media, TNB people create linguistic indicators of gender dysphoria, which NLP can transform into independent variables for ML models. By mining text to extract independent variables, NLP transforms these linguistic indicators of gender dysphoria into scores representing psychological factors in language use (ie, psycholinguistic attributes [30]), ecologically valid dictionaries of semantic and syntactic descriptions of gender dysphoria used among TNB people (ie, keywords and n-grams [31]), numerical estimations of the degree to which the social media content is pleasant or unpleasant (ie, emotional valence and sentiment lexicons [31]), composite scores of whether psychological distress is evident (ie, depression, anxiety, stress, and suicidal ideation [DASS] [32]), and text-to-number transformations that capture how words are used in context (ie, word embeddings [33]). If NLP-generated independent variables are predictive of psychological constructs, models with NLP-generated independent variables can be used to tailor mental health care interventions and applications to the specific needs of TNB people [23,34]. For example, the type of intervention content displayed can change based on whether gender dysphoria is detected in social media text.

Fourth, social media—particularly websites such as Reddit (Reddit Inc)—enable anonymous posting of highly sensitive content. Research indicates that Reddit users may use *throwaway* accounts to hide their real-life identity while posting emotionally evocative and deeply personal details about their mental health [35]. For a stigmatized and marginalized group such as TNB people, Reddit may provide an optimal, publicly available window into struggles with gender dysphoria.

Fifth, unlike traditional statistical methods, ML models rely on fewer statistical assumptions, can estimate relationships without a priori theoretical model specification, and can maintain hundreds of predictors simultaneously [34]. One of the benefits of these methodological properties is that ML models, in comparison with traditional statistical models, improve the predictive accuracy of both clinical (eg, nonsuicidal self-injury [36]) and TNB-specific (eg, minority stress [37,38]) psychological constructs. Another benefit of ML models, compared with traditional statistical models, is their flexibility and responsiveness to the emergent predictors of gender dysphoria. That is, if ML is used in downstream technology-delivered interventions for gender dysphoria, these methodological properties would facilitate web-based, real-time adaptation to the community- and person-specific ways in which TNB people experience and describe gender dysphoria.

### Purpose of This Study

In recognizing technology-delivered interventions for gender dysphoria as an important next step in optimizing care for TNB people, this study investigated the preliminary effectiveness of using NLP-generated independent variables to model gender dysphoria. The modeling objective was framed as a classification task whereby we sought to predict whether gender dysphoria was present or absent in a social media post. Multiple ML models were tested to identify the best-performing algorithm for modeling gender dysphoria on social media, including an examination of which NLP-generated independent variables contributed the most to accurate and inaccurate predictions.

## Methods

### Data Collection and Procedure

The analyses were part of a larger study on using ML and NLP to identify minority stress among sexual and gender minority individuals via social media posts. Reddit data posted on TNB-specific forums were downloaded using the Pushshift application programming interface and BigQuery (Google LLC). Reddit posts were randomly sampled from subreddits related to sexual and gender minority people (eg, r/gay and r/trans; n=1000) or downloaded from the subreddit r/GenderDysphoria (n=1099). The demographic characteristics of the users were not available on Reddit and not inferred using ML methods to protect user anonymity and in light of ethical concerns around automated gender inference [39]. The Reddit posts were cleaned by removing special characters and nonword formatting (eg, URLs) using software.

After cleaning the data, a qualitative, a priori content analysis was performed to establish the initial data set (N=2099 [40]). The qualitative team (first through fifth, eighth, and ninth authors) comprised sexual and gender minority clinicians, researchers, and students with formal training in content analysis and clinical experience with TNB clients; this team included both cisgender and TNB individuals. The qualitative team established a gender dysphoria codebook from previous research (Table S1 in Multimedia Appendix 1 presents the codebook [2,38,41]). Specifically, to be labeled a positive example of gender dysphoria, posts had to (1) show evidence of at least 1 manifestation of gender dysphoria (eg, a strong desire for a different gender, a strong conviction in being a different gender, perceived incongruence between sex and gender, dislike for socially assigned gender identity, or “feeling dysphoric”) and (2) demonstrate at least 1 clear negative consequence of gender dysphoria (ie, distress or impairment); this is elaborated in Table S1 in Multimedia Appendix 1.

Pairs of coders applied the codebook to small sets of Reddit posts (n=25), met weekly to discuss discrepancies, and revised the codebook until consensus was reached (ie, Cohen κ≥0.80 or simple percent agreement reached ≥0.90 [42]). After this training phase, all members of the qualitative team (except the first author) coded the remaining Reddit posts independently. The first author kept analytic memos, facilitated group discussions, and randomly audited 500 posts to increase credibility and trustworthiness [43]. During auditing, in cases where the first author disagreed with an independently coded post, the first author’s coding was used. As only the first author randomly audited posts after the initial training phase, interrater reliability was not calculated again.

A total of 791 positive examples of gender dysphoria were coded. As ML models benefit from balanced data (ie, a 1:1 ratio of positive and negative examples of a clinical construct), the positive examples were matched with a random selection of 791 negative examples (ie, posts displaying no gender dysphoria); 7 negative examples were dropped because the posts contained <10 characters. Thus, the final data set contained 1573 posts and remained substantially balanced. Table 1 shows examples of Reddit posts with and without gender dysphoria. The arithmetic mean length of the posts was 201 words (SD 205, median 141, range 2-2116). Figure S1 in Multimedia Appendix 1 provides a visualization of the distribution of word count in the Reddit posts.

**Table 1:** Qualitative examples of coding Reddit posts for the presence or absence of gender dysphoria.^a^

Gender dysphoria	Reddit post
Yes (1)	“[...] I do not want to be perceived as a female [...] I totally pass [...] I do not want to be embarrassed [...] introducing myself as they/them/theirs [...] I have learned to deal with my breasts [...]”
Yes (1)	“[...] I hate being a boy so much [because] looking at my genitalia makes me so uncomfortable [...]”
Yes (1)	“Always wanted breasts and feminine features [...] have a huge fear of having [...] masculine features [...] I get so uncomfortable”
Yes (1)	“I have been feeling horrible about myself [...] I will never be taught how to [wear] makeup like [cisgender girls...] my parents refuse to use my pronouns and name [...] I hate every waking moment [...] I look like shit and I will never pass as a woman [...] cursed to be born in this [male] body”
Yes (1)	“I feel gender dysphoria/disconnect with my assigned gender (AFAB) [...] I get so grossed out when I do something in a girly way”
No (0)	“[...] always thought that my fantasies of being a woman [...] have been a fetish [... because] I only wanted to be a woman when I am horny [...]”
No (0)	“[...] I’m starting to think that I’m gay [...] maybe I’m more attracted to women now [...] I’m worried I might be gay.”
No (0)	“[...] married for two years [...] with a kid and [my husband came out as transgender but] I love him very much [...] can anyone relate?”
No (0)	“[Here’s some art] I made to show my friends [...]”
No (0)	“[...] my wife and I have been married for 8 years [...but I have a lot of free time due to COVID-19, so I have] built a patio, completely rearranged our living room [...]”

^a^Text has been cleaned, truncated (ie, “[...]”), and slightly edited for readability and to protect the confidentiality of Reddit users.

### Measures

NLP techniques generated 949 independent variables, which are organized into 6 categories; these are described in the following subsections, starting with the simplest and ending with the most complex ones.

#### Psycholinguistic Attributes

Of the 949 independent variables, 93 (9.8%) were extracted from the Linguistic Inquiry and Word Count (LIWC) lexicon [30]. LIWC is a propriety software that uses a dictionary-based approach to generate psycholinguistic attributes of text data. The LIWC dictionary was developed from emotion rating scales and common English words, using the consensus of expert judges; this dictionary has been extensively adopted to conduct psycholinguistic analyses on social media data [44]. LIWC-generated independent variables related to general summary variables (eg, overall emotional tone), linguistic dimensions (eg, pronouns), grammatical structure (eg, the presence of common verbs), and psychological processes (eg, concerns about work, drives for power, cognitive processes, and mentions of social relations) were created.

#### Clinical Keywords

Given that TNB people are typically required to interface with health care systems to obtain a diagnosis of gender dysphoria and receive specialized medical care (eg, gender affirmation surgery [6]), TNB populations may use clinical language to describe their experiences with gender dysphoria. Thus, text from the *Diagnostic and Statistical Manual of Mental Disorders, Fifth Edition* (*DSM-5*) chapter on gender dysphoria was mined for independent variables. The *DSM-5* text was tokenized (ie, transformed into words), stemmed (eg, “assignment” → “assign”), and cleaned before extracting the top 5 clinical unigrams (ie, dysphori*, natal, disorder, assign*, and develop*). Regular expressions detected the presence of at least 1 unigram to generate a single clinical keyword independent variable, where 1 indicated that a clinical keyword was present, and 0 indicated that the keywords were absent.

#### n-Grams

To identify the unique linguistic content associated with gender dysphoria, 550 n-grams (ie, word units of size *n*; n=1, 2, and 3) were generated. The first author audited the 250 unigrams, 250 bigrams, and 50 trigrams extracted to ensure that the n-grams were not related to off-topic terms (eg, “lockdown” and “gift card”). The 250 unigrams, 250 bigrams, and 50 trigrams were selected because higher numbers yielded less unique n-grams (ie, words that could be related to any clinical construct), and lower numbers missed important n-grams related to gender dysphoria (eg, “flat male chest”). Past research demonstrates that n-grams are excellent predictors of psychological constructs on social media platforms [45]. Reddit posts were grouped according to the presence (1) or absence (0) of gender dysphoria. The term frequency–inverse document frequency (TF-IDF), a measure of how important an n-gram is in defining a group of text, was calculated. Higher TF-IDF scores indicate that a given n-gram is more important in distinguishing one group of text from another group of text [31]. After calculating TF-IDF scores, positive examples of gender dysphoria were selected, and the top 550 n-grams with the highest TF-IDF scores were extracted as independent variables.

#### Emotional Valence (Sentiment)

One independent variable related to the overall degree of positive or negative sentiment (ie, emotional valence) was created from 2 sentiment lexicons. AFINN [46] is a lexicon in which formal English words are associated with an integer representing positive or negative sentiment. For example, words such as accept, curious, and laugh are scored as +1, and words such as ambivalent, hide, and rejected are scored as –1. Words with greater emotional intensity are scored accordingly (eg, “hate”=–3 and “yummy”=+3). The slangSD [47] lexicon functions similarly to AFINN but was established for informal English words often used on social media (eg, “hey bitches!”=+1 and “adulting”=–1). Reddit posts were tokenized and merged with AFINN [46] and slangSD [47] sentiment lexicons to generate a score for each word within a Reddit post. Words without AFINN and slangSD matches were scored 0. The score of each word ranged from –5 (extremely negative) to +5 (extremely positive). For each Reddit post, the AFINN-slangSD scores were summed to yield a total score for the Reddit post, where lower negative scores indicated more overall negative sentiment, higher positive scores indicated more overall positive sentiment, and scores close to 0 represented an overall neutral sentiment. Total sentiment scores ranged from –960 to +96.

#### Psychological Distress (DASS)

Given that a diagnosis of gender dysphoria requires the presence of distress [2], independent variables related to DASS were generated by replicating an approach used in past research [32]. This approach involves 4 support vector machine (SVM) classifiers trained for each psychological condition. Positive examples of each psychological construct included Reddit posts from r/depression, r/anxiety, r/stress, and r/suicidewatch. Negative examples were posts from the 10 most popular subreddits (eg, r/askscience, r/movies, and r/aww). Independent variables for the SVM classifier were generated from 5000 n-grams (n=1,2, and 3). Each data set was balanced. The independent variables generated from this DASS approach are considered clinically relevant by mental health professionals [38]. Refer to GitHub for more details [48] and Table S3 in Multimedia Appendix 1 for performance metrics from training the DASS classifiers. After training the classifiers, they were applied to the current data set to classify each post as evincing linguistic markers of depression, anxiety, stress, and suicidal ideation (1=at least 1 DASS construct is present) or not (0=no DASS construct is present). These predicted classes were used as independent variables in the ML models.

#### Word Embeddings

Word embeddings are a means to represent words as vectors (numerical representations) in a high-dimensional (eg, 300) latent lexicosemantic space such that vectors of lexicosemantically similar words are closer to each other. Word embeddings were generated using Word2Vec [33], a word mapping program trained on 100 billion words from Google News (Google LLC). Word2Vec was used to transform each word in a Reddit post into a 300-dimensional vector (eg, the post “I hate my body so much” was transformed into six 300-dimensional vectors). For each Reddit post, the average word embedding was calculated using the element-wise arithmetic mean of each 300-dimensional vector generated from individual words within the Reddit post. Each word embedding dimension was added to the ML models as an independent variable.

### Data Analytic Plan

Both R (R Foundation for Statistical Computing) and Python (Python Software Foundation) were used to analyze the data. All ML models were implemented using the Python package *scikit-learn* [49]. All analytic scripts are publicly available [48].

A total of 6 ML models were tested. Testing and comparing the performance of multiple ML models are necessary to identify the optimal algorithm because each algorithm differs in how it generates model parameters [50,51]; this difference varies by classification task [28,38]. The models were selected because of their well-established performance in classifying psychological constructs from social media text [27,28,52]. Although a full description of each ML model is beyond the scope of this paper (the study by Raschka et al [50] provides a thorough introduction), this paper provides a general outline of each model and its hyperparameters (ie, options the researcher sets before estimation).

First, we tested a decision tree, an algorithm which classifies Reddit posts using a tree-like flow diagram. Independent variables providing the greatest information gain in the classification decision lie at the root of the decision tree, and Reddit posts are passed down through the tree structure using conditional logic. The entropy impurity function was used, and prepruning was executed by setting the maximum depth to 10. Second, we tested a linear SVM, which separates positive and negative examples of gender dysphoria by maximizing the margin between 2 hyperplanes. A squared L2 penalty of 1 was added to reduce overfitting. Third, a multiple logistic regression classifier was trained with an L2 penalty of 1, and 100 iterations were executed. Fourth, a naive Bayes classifier, which applies Bayes’ theorem to determine the probability of gender dysphoria being present given the values of a Reddit post’s independent variables [53], was trained and tested. The prior probabilities were not specified ahead of time (ie, they were data driven), and variance smoothing was set to 1^–9^. Fifth, a random forest classifier was used. A random forest randomly samples the independent variables and assembles simple decision trees to classify a Reddit post via majority voting [50]. A random forest was executed with 100 decision trees, an entropy impurity function, and a maximum forest depth of 10. Finally, an extreme gradient boosting (XGBoost) classifier was trained and tested. XGBoost, similar to random forest, creates an ensemble of decision trees and random samples of features, but the decision trees are executed through different boosting iterations, wherein the error from one boosting iteration is used to inform the next boosting iteration [54]. The learning rate was set to 0.1, and the maximum depth was set to 10.

The data set (N=1573) was split into training (n=1258, 79.97%) and test (n=315, 20.03%) sets to determine model performance. During the training of each of the 6 ML models, k-fold cross-validation (k=10), a robust approach to model selection and error estimation [50], was used to reduce the random effect of splitting the training set into a validation set. We compared the accuracy (ie, the proportion of correct predictions), precision (ie, the number of true positives divided by the sum of true positives and false positives), recall (ie, the number of true positives divided by the sum of true positives and false negatives), *F*_1_-score (ie, the harmonic mean of precision and recall), and area under the receiver operating characteristic curve (AUC) to evaluate model performance.

After testing and comparing the performance of these 6 ML models, we selected the top-performing 2 models and optimized their hyperparameters with random search to understand which independent variables best predict gender dysphoria. A random search randomly selects hyperparameter values from distributions of possible values; executes the ML model multiple times; and, during each execution, uses a different set of hyperparameters. Random search addresses the limitations (eg, resource intensive) of manual hyperparameter tuning [50]. The hyperparameter set yielding superior model performance was retained. To find the most informative independent variables, we used feature selection techniques (refer to the study by Chandrashekar and Sahin [55] for a review), including the automatic feature selection used in random forests and XGBoost.

Consistent with ML approaches to analyzing social media text [38], 2 techniques of error analysis were applied to test the results of the best-performing ML model. First, a confusion matrix was computed to identify false positives (ie, predicting gender dysphoria when it was absent) and false negatives (ie, predicting no gender dysphoria when it was present). These were then analyzed using a qualitative content analysis [40] to identify the linguistic characteristics of the misclassified Reddit posts. The first author performed the content analysis of all misclassified Reddit posts, and the third author audited the content analysis to increase credibility [43]. Second, an error tree was calculated to find the independent variables that contributed the most to the errors [41]. The error tree predicted whether the best-performing model classified a Reddit post correctly (1) or incorrectly (0). If the fidelity (1–|actual accuracy of the best model–estimated accuracy of error tree|) of an error tree is ≥0.90, then the error tree is representative of the best-performing model (ie, conclusions about the error of the independent variables can be drawn [41]). In an error tree with adequate fidelity, each leaf node partitions the error into smaller subsets with similar features and similar performance, which enables the identification of the most problematic independent variables.

### Ethical Considerations

The institutional review board of the first author’s institution approved this study as exempt research. As such, the study proceeded without obtaining informed consent from TNB Reddit users. Moreover, Reddit users who authored posts in the data set were not compensated because the data were publicly available. The preprocessed, deidentified data were not released publicly to protect TNB Reddit users. Because a deidentified NLP-based data set could potentially be reverse engineered by motivated, malicious actors attempting to target marginalized TNB people on the web, especially during the rampant and ongoing assaults on TNB rights in the United States [56,57], we have restricted access to the deidentified data set to qualified researchers who request access. This study was not preregistered.

## Results

### ML Model Training

The results from training each ML model are presented in Table 2. The ensemble approaches (ie, random forest and XGBoost) yielded superior performance, as evidenced by higher accuracy, precision, recall, *F*_1_-score, and AUC scores relative to the other classifiers. Thus, hyperparameter tuning with random search was executed on random forest and XGBoost classifiers; the results are presented in Table 3. The best-performing random forest classifier had a maximum depth of 56, sampled 62.1% (586/949) of the independent variables, and used 391 estimators to achieve an accuracy of 0.82 at test time. The best performing XGBoost classifier used a learning rate of 0.1742, an L2 penalty of 3, a maximum depth of 33, and a subsample of 55.74% (529/949) of the independent variables to achieve an accuracy of 0.83. Moreover, XGBoost ran in 1.23 seconds, whereas random forest ran in 29.35 seconds. As XGBoost performed slightly better and ran approximately 24 times faster than random forest, XGBoost was retained for the remaining analyses.

Notably, our XGBoost performance metrics on the held-out test set were slightly greater than those on the held-out training set (Table 3), which is unusual but not impossible. Nonetheless, we verified the findings by resplitting the data set into new training and test sets. Using the tuned hyperparameters, we re-executed the XGBoost algorithm and obtained the following performance metrics: accuracy=0.84, precision=0.86, recall=0.80, *F*_1_-score=0.83, and AUC=0.84. Although executing XGBoost on new data would have been preferred, additional data were unavailable, and these recomputed metrics were comparable with our original metrics, thus increasing the confidence in our results.

**Table 2:** Performance during model training without hyperparameter tuning.^a^

Model	Performance metrics				
	Accuracy, mean (SD)	Precision, mean (SD)	Recall, mean (SD)	*F*_1_-score^b^, mean (SD)	AUC^c^, mean (SD)
Decision tree	0.70 (0.03)	0.70 (0.07)	0.70 (0.05)	0.70 (0.03)	0.71 (0.03)
Support vector machine	0.75 (0.05)	0.79 (0.04)	0.68 (0.07)	0.73 (0.05)	0.82 (0.04)
Logistic regression	0.77 (0.05)	0.80 (0.04)	0.71 (0.06)	0.76 (0.04)	0.85 (0.04)
Naive Bayes	0.80 (0.05)	0.94 (0.03)	0.64 (0.06)	0.76 (0.05)	0.80 (0.04)
Random forest	*0.80* (0.04)^d^	*0.80* (0.04)	*0.81* (0.03)	*0.81* (0.03)	*0.87* (0.04)
XGBoost^e^	*0.80* (0.03*)*	*0.80* (0.04)	*0.82* (0.03)	*0.81* (0.02)	*0.88* (0.04)

^a^During training, k-fold cross-validation (k=10) was used; hence, the arithmetic mean is shown along with the SD in parentheses.

^b^*F*_1_-score is the harmonic mean of precision and recall.

^c^AUC: area under the receiver operating characteristic curve.

^d^Italicized values indicate the best-performing models.

^e^XGBoost: extreme gradient boosting.

**Table 3:** Performance during model training and testing using random search to tune hyperparameters.

Model			Performance metrics								
			Accuracy		Precision		Recall		*F*_1_-score^a^		AUC^b^
**Training, mean (SD)^c^**											
	Random forest	0.82 (0.05)		0.83 (0.06)		0.81 (0.06)		0.82 (0.05)		0.88 (0.03)	
	XGBoost^d^	0.82 (0.03)		0.82 (0.06)		0.82 (0.03)		0.82 (0.03)		0.89 (0.03)	
**Testing^e^**											
	Random forest	0.82		0.82		0.82		0.82		0.82	
	XGBoost	*0.84* ^f^		*0.83*		*0.85*		*0.84*		*0.84*	

^a^*F*_1_-score is the harmonic mean of precision and recall.

^b^AUC: area under the receiver operating characteristic curve.

^c^During training, k-fold cross-validation (k=10) was used; hence, the arithmetic mean is shown along with the SD in parentheses for the training metrics.

^d^XGBoost: extreme gradient boosting.

^e^The testing metrics are single values because the metrics were calculated once.

^f^Italicized values indicate the best-performing model.

### Most Informative NLP-Generated Independent Variables

To identify the most informative independent variables, predictors retained by the XGBoost classifier were used because XGBoost, similar to other boosted decision trees, automatically selects the most informative independent variables [54,58]. Table 4 depicts the independent variables retained by category and the relative importance of each independent variable category.

Of the 949 independent variables generated using NLP, the XGBoost classifier retained 396 (41.7%) to use as predictors of gender dysphoria. On average, clinical keywords were substantially more predictive of gender dysphoria on Reddit than other independent variable categories.

Independent variables from the clinical keyword, psycholinguistic attribute, and word embedding categories constituted the top 10 most informative predictors in the model. The information gained by the top 10 most informative independent variables ranged from 5.07 (using uncommon punctuation, such as emojis) to 33.42 (using *DSM-5* clinical keywords, such as “dysphoric”). That is, in modeling gender dysphoria on social media, the most informative independent variables were (1) syntactic permutations of 5 clinical keywords (eg, dysphoria, natal, disorder, assignment, and developing); (2) 4 psycholinguistic attributes, including the use of exclamation marks, a discussion of the body, a discussion of power and social influence, and the use of uncommon punctuation; and (3) 5 specific dimensions of a 300-dimensional word embedding. Table S2 in Multimedia Appendix 1 provides a full list of features ranked by importance.

**Table 4:** Independent variables selected by extreme gradient boosting (XGBoost) as important predictors of gender dysphoria.

NLP^a^-generated independent variables	Total variables (N)	Variables selected (n)^b^	Information gained^c^
Clinical keywords	1	1	33.4
Sentiment	1	1	2.51
Word embeddings	300	297	1.66
Psycholinguistic attributes	93	90	1.56
DASS^d^	4	3	1.41
n-Grams	550	4	0.55

^a^NLP: natural language processing.

^b^Variables selected by XGBoost to retain [58].

^c^The information gained was calculated using the Gini index [50,58].

^d^DASS: depression, anxiety, stress, and suicidal ideation.

### Misclassification Analysis

Figure S2 in Multimedia Appendix 1 presents the confusion matrix of test data and examples of false positives and false negatives. Content analysis of the 16.2% (51/315) misclassified test examples in the data set yielded 10 nonexclusive categories of misclassification. When a Reddit post was misclassified by XGBoost, the post (1) expressed uncertainty, confusion, or ambivalence about the person’s experience, such as disclosing dysphoric feelings but then denying them (24/ 51, 47.1% misclassified posts); (2) featured a stressful experience unrelated to gender dysphoria, such as experiencing prejudice based on the person’s sexual orientation (18/51, 35%); (3) was incorrectly coded by the qualitative team (14/51, 28%); (4) expressed insufficient linguistic and semantic markers of gender dysphoria, such as mentioning “bottom dysphoria” once and then describing another experience in full detail (13/51, 26%); (5) described experiences that primarily occurred in the past (7/51, 14%); (6) showed evidence of identity exploration (6/51, 12%); (7) expressed aspects of human sexuality, such as sexual attraction or sex-negative beliefs (eg, “sex is gross”; 5/51, 10%); (8) described socially based gender dysphoria, such as emphasizing the style of dress (4/51, 8%); (9) expressed strong affective or cognitive reactions to an experience unrelated to gender dysphoria (4/51, 8%); or (10) discussed body image concerns, such as body dysmorphia (3/51, 6%). The top misclassification category for false negatives was expressions of insufficient linguistic and semantic markers of gender dysphoria (12/51, 24% of the posts). The top misclassification category for false positives was expressions of uncertainty, confusion, or ambivalence about the person’s experience (16/51, 31%). Note that percentages do not add up to 100 because a Reddit post could have more than one misclassification category applied.

Building on the content analysis to comprehend errors, we computed an error tree. The fidelity of the error tree was 0.99, suggesting that the error tree was representative of the XGBoost classifier. Figure S3 in Multimedia Appendix 1 presents the top 10 independent variables associated with misclassifications in the error tree. Word embedding dimensions (ie, w2v_297, w2v_47, etc) and psycholinguistic attributes (ie, differ and QMark) were most associated with error. As an example, consider the independent variable most associated with misclassification error (ie, “differ,” as presented in Figure S3 in Multimedia Appendix 1). Error tree results indicate that the cognitive process of contrasting one idea from another (eg, indicated by words such as “but” or “hasn’t” [30]) is most likely to cause a wrong prediction when its standardized value is between –0.9 and 0.4.

## Discussion

### Principal Findings

This study investigated the preliminary effectiveness of modeling gender dysphoria on social media using ML models and NLP-generated independent variables, an important first step in incorporating ML and NLP in technology-delivered interventions [23] for gender dysphoria. Evidence indicates that an ML and NLP approach produced highly accurate classifications of social media content with and without linguistic indicators of gender dysphoria, thereby contributing to the growing evidence on the importance of incorporating ML and NLP designs in clinical science [24,28,32], especially when studying understudied and marginalized populations [37,38]. Ensemble ML models yielded superior performance, with XGBoost modeling gender dysphoria slightly better and substantially faster than random forests; these gains in accuracy and speed are vital for the real-time deployment of ML in technology-delivered interventions [59]. Incorrect prediction of gender dysphoria was minimal, with misclassifications driven by unique semantic and syntactic features.

Misclassifications were evident in social media posts that expressed uncertainty, general stress, insufficient linguistic content, past-tense language, aspects of human sexuality, socially based gender dysphoria, strong reactions to non–dysphoria-related experiences, and body image concerns. The error tree results overlapped with the results of the qualitative analysis of misclassification error because expressions of uncertainty were likely transformed into psycholinguistic attributes related to differentiation (ie, using words such as “but” to differ thoughts) and questioning (ie, using question marks [30]). Although uncertainty about one’s gender identity is a prominent theme in the phenomenology of gender dysphoria [60], uncertainty about whether one is experiencing gender dysphoria resulted in TNB and gender-questioning people providing evidence for and against gender dysphoria, which the ML models could not adequately untangle. In technology-delivered interventions, expressions of uncertainty could result in erroneous suggestions for intervention content aimed at reducing gender dysphoria, which, for a TNB person who is unsure about their gender dysphoria, could exacerbate confusion.

Other misclassifications have implications for technology-delivered interventions. The use of past-tense language, for example, detracted from the predictive accuracy of the models; this is consistent with other ML and NLP research on the psychological constructs pertinent to the well-being of TNB people [38]. Predicting past instances of gender dysphoria is less useful because it suggests a TNB person may no longer need intervention. If a mobile health app suggests intervention content related to gender dysphoria to a TNB person who merely recalled their past experiences of gender dysphoria with a friend, the app could be perceived as invalidating. Misclassification of posts with socially based gender dysphoria or strong reactions to non–dysphoria-related experiences might derive from unbalanced data along these 2 dimensions. For example, misclassification of posts with strong reactions to other stimuli may be an artifact of a data set with many linguistic indicators of strong negative affect (ie, negative sentiment, which ranged to –960, was significantly more prominent than positive sentiment, which ranged to +96). Similarly, misclassification of posts with socially based gender dysphoria might reflect the dominance of text content related to body-based gender dysphoria versus gender dysphoria resulting from incorrect pronoun use. Thus, more research is necessary before incorporating the results from this study into technology-delivered interventions.

As with existing research [36], this study examined predictor importance, with 2 notable highlights. First, clinical keywords from the *DSM-5* (eg, “dysphoric”) were the most associated with the modeling of gender dysphoria on social media. Many TNB people on the web discuss their gender dysphoria using clinical terms, which could reflect the need for TNB people to educate providers who lack competence in the health of TNB people [61], the historical medicalization of TNB identities [62], using clinical terms to access health care [6], or some combination. Second, the XGBoost model removed most n-grams during estimation, yet as shown in Table 4, n-grams were the second most important predictors of gender dysphoria. Given that other NLP and ML approaches using n-grams also leverage millions of data points [45], it is possible that the existing data set was too small to find an adequate number of n-grams with sufficiently high TF-IDF scores. Future research using larger data sets is necessary to confirm predictor importance because, from an applied perspective, having a valid set of predictors (as opposed to many noisy predictors) could reduce the computation time of ML models—an important consideration in on the web, real-time technologies [59].

### Considerations for Incorporating the Findings Into Technology-Delivered Interventions

Although future research should adequately address misclassifications and confirm predictor importance, this study provided strong initial evidence (ie, all performance metrics were ≥0.80) of modeling gender dysphoria from social media using ML and NLP. The present findings can be used in smartphone apps, private practice management software, patient portals for semiautomatic preliminary diagnoses, and just-in-time reminders to use behavioral coping tools. For instance, a clinician and TNB client could agree to install an app on the client’s smartphone that uses our ML and NLP approach to analyze text; predict whether gender dysphoria has re-emerged after substantial remission; and subsequently trigger automated intervention-related messages, which were uploaded in advance to a server by the clinician and encourage the implementation of the skills learned in therapy. Alternatively, an app with prerecorded gender-affirming interventions for TNB people unable to see a clinician could be used, with intervention content triggered as a smartphone notification when expressions of gender dysphoria are detected. Another example might be supporting clinical decision-making: eventually, TNB clients could connect their social media to their trusted therapist’s practice management software, and, if our ML models detected gender dysphoria, the therapist could be alerted to offer a psychotherapy session and discuss gender dysphoria with the client. On the basis of our findings, such technologies could serve as low-cost, alternative, easily accessible interventions for TNB people living in rural areas or for TNB people wary of provider discrimination in face-to-face patient care (eg, TNB people living in states with substantial anti-TNB legislation).

Of course, before our ML- and NLP-based models can be integrated into real-world interventions, the issues of gender dysphoria base rates and data imbalance must be addressed. Aside from a couple of studies [1,63], the base rate of gender dysphoria in TNB people is largely unknown. Estimates of the prevalence of gender dysphoria in TNB children range from 33% [63] to 78% [1], with some evidence that the levels of gender dysphoria change over time [63]. Although balancing the data before model training was reasonable in light of these imprecise prevalence estimates of gender dysphoria, data balancing also improves ML modeling by largely sidestepping the problem of imbalanced data [64]. Balancing data can be problematic when ML and NLP models are integrated into technology-delivered interventions because if the base rate of gender dysphoria is not 50% (which we assumed by balancing the data), then the models may make incorrect predictions (eg, suggesting intervention content to a TNB person who is *not* experiencing gender dysphoria) when deployed in real-world settings. In other words, if the portion of social media posts showing evidence of gender dysphoria during ML model training does not match the base rate of gender dysphoria in the real world, harmful false positives may occur [65]. Thus, more research is needed to understand the prevalence of gender dysphoria among TNB people, and future ML- and NLP-based studies should use other techniques for handling imbalanced data [64].

The possibility of incorrect predictions raises the point that simpler, noncomputational technology-delivered interventions for gender dysphoria treatment may be favorable alternatives. Survey-based approaches leveraging measurement-based care (ie, basing clinical services on client data throughout treatment [66]) could be integrated into technology-based interventions for TNB people. For example, a mobile app could intermittently assess symptoms of gender dysphoria using a questionnaire and then modify the delivery of intervention content based on survey scores. However, in comparison with passive sensing approaches such as the ML and NLP models presented in this paper, a measurement-based care approach using survey assessment of gender dysphoria may be less equipped to solve the personalization (ie, frustration that digital health apps are not tailored to individual concerns and symptoms) and engagement (ie, low motivation to use digital health apps) limitations of technology-delivered interventions [67,68]. Ultimately, choosing one approach over the other may be inferior to combining them. Mental health care is moving toward automating measurement-based care using ML and NLP [69] and, in turn, using traditional measurements such as surveys to fine-tune the predictions of ML- and NLP-based passive sensing.

The integration of our ML and NLP results into technology-delivered interventions would require the resolution of several important challenges. First, researchers would need to decide where the ML and NLP models would run [70,71]. Running the ML and NLP models on a TNB person’s phone might improve privacy but may exceed the phone’s computational power. Alternatively, transmitting social media data to the cloud might expand access to computational power, but data loss in transmits could result in significant data availability bias [72]. Second, control and customization are important in designing technology-delivered interventions for this population [73]. Researchers are encouraged to think carefully about which social media data (eg, which websites and what type of content) would be used if the ML and NLP models could run on limited text data, and what automated intervention content might be impaired if a TNB user decides to turn off data sharing capabilities. Third, the methods we report in this paper, although promising, rely on technology that may become obsolete in the next decade [74,75], thus requiring researchers to conduct new validation studies and adjust their computational architecture to keep their technology-delivered interventions up to date. In summary, researchers must consider these challenges, as well as others, before incorporating our ML and NLP models into technology-delivered interventions for gender dysphoria.

### Ethical Implications

Despite the potential clinical benefits of ML and NLP approaches to modeling gender dysphoria, unintended harm must be considered. A thorough feasibility and acceptability study of ML and NLP for TNB-specific health care is required to ensure that TNB people (1) endorse such research and (2) desire the proposed clinical applications. The design of such clinical technologies should, ideally, include TNB people in key roles throughout the design-to-deployment pipeline or, at a minimum, use TNB advisory boards to guide clinical applications [76]. Including TNB individuals in the research-to-app-development pipeline may reduce the likelihood of unintentional transphobic or otherwise harmful messaging.

Furthermore, additional model optimization is likely required before the findings can be applied in clinical settings. For instance, although our findings are highly accurate, there remains a substantial margin of error and unclear base rates, as described earlier. In technology-delivered interventions, incorrectly classifying a piece of text as showing evidence of gender dysphoria could trigger intervention content that does not resonate with the TNB client, prompting the client to stop using the technology even if the technology-delivered intervention, overall, yields substantial clinical benefit [77]. Moreover, the potential use of these findings by malicious actors cannot be overstated. Motivated anti-TNB political actors could use an ML and NLP approach to target susceptible TNB youth experiencing gender dysphoria, redirecting them to conversion therapy or outing them to their caregivers. In political environments where TNB people face state-sponsored persecution, state powers could use our findings as surveillance methods to estimate whether a citizen is TNB, thereby increasing the risk of harm. In light of these possible negative political outcomes, although we provide all analytic scripts associated with the current project on GitHub [48], we have not shared the text data from Reddit to minimize the possible identification of TNB Reddit users.

### Limitations

There are limitations to this study. First, the analyses were restricted to text data from 1 social media platform (ie, Reddit), which is limiting because there are demographic differences in social media website use (eg, Reddit users are primarily White and Hispanic [78]), and communication options vary by platform (eg, Reddit users can write paragraphs per post, whereas Twitter users are limited to 280 characters per post). In addition, the anonymity affordances of Reddit “throwaway” accounts (eg, substantial disinhibition [79] and highly personal details about one’s mental health distress [35]) may have resulted in more disclosures of gender dysphoria, which might not extend to other social media, such as Facebook, where the expectation for the accurate representation of one’s real-life identity is enforced. As the findings of this study may not be generalizable to different platforms, future research should validate the ML models across other social media websites.

Second and relatedly, Reddit posts were gathered from specific web-based communities, or “subreddits,” for TNB populations (eg, r/trans). TNB people who post on these communities likely differ from those who do not seek TNB-specific web-based forums. Although the influence of subreddit-specific community norms was minimized by sampling from multiple subreddits, it remains unknown whether the algorithms examined in this study would detect gender dysphoria across the Reddit ecosystem. Future work might first test optimized ML models on subreddits for sexual minority people (eg, r/bisexual), followed by subreddits for schools (eg, r/ucla) or local communities (eg, r/Bronx).

Third, we had no way of verifying the TNB identity of all Reddit users in our data set. Although many Reddit users announced their identity at the beginning of their posts, this behavior was not uniform. It remains possible that non-TNB people (eg, bots and “trolls”) may have authored some posts in our data set.

Fourth, because a random search was used, not all possible hyperparameters were tested for the ML models, so performance improvements may be possible. Future researchers might consider using a grid search to tune hyperparameters.

Fifth, regarding features, at least 1 word count–sensitive feature was used. Emotional valence was calculated as a mean score. In longer posts, where there are more opportunities to express negative or positive sentiment, a mean score (vs a median score or a normalized score) could skew the feature. In other words, longer posts are more likely to be negative or positive simply because there are more opportunities to express positive or negative sentiment. Future researchers should consider normalizing, or correcting, word count–sensitive features (eg, dividing the feature score by the length of the post). Nonetheless, because the emotional valence feature was the 132nd most important feature in the XGBoost algorithm and considering the facts that the average length of the posts was relatively short and the distribution of word counts was relatively condensed below approximately 250 words, we believe that our mean-scored emotional valence feature is defensible if not optimal.

Sixth, deep neural networks, such as sequence-based algorithms (ie, recurrent neural networks), were not tested. The decision not to examine neural networks was due to hierarchical latent representations’ ability to compromise interpretability (ie, researchers do not know how the model determines the outcome [50]). Nonetheless, because neural networks often yield superior predictive performance in comparison with the ML models investigated in this study [80], future researchers should examine the utility of deep neural networks in predicting gender dysphoria. Indeed, recurrent neural networks may be poised to overcome the sources of misclassification error identified in this study due to the addition of hidden, nonlinear layers.

Finally, the results from ML models depend on high-quality data [50]. As Reddit posts were coded for the presence of gender dysphoria and error analysis revealed some incorrectly coded posts, model performance may be limited by the qualitative coding process. Although the researchers sought to minimize coding error using quality assurance measures (eg, auditing), future research might use social media to either predict scores on a psychometric measure of gender dysphoria or distinguish TNB people with a clinician-derived diagnosis of gender dysphoria from TNB people with no such diagnosis.

### Conclusions

By modeling gender dysphoria on social media using ML and NLP among a community sample of TNB people, this study constitutes a crucial, preliminary step toward creating technology-delivered interventions for this marginalized population. ML models powered by NLP-generated independent variables may one day be part of the driving infrastructure for automating evidence-based treatments for TNB people. Although automation cannot and should not replace trained psychologists and therapists, automated prevention and intervention efforts can supplement formal psychotherapy treatment and increase health equity by providing low-cost, scalable care alternatives for TNB people facing barriers to care, as well as help identify and address gender dysphoria among TNB individuals awaiting more intensive, personalized care.

## Appendix

Multimedia Appendix 1 Supplementary tables and figures.

## Abbreviations

AUC: area under the receiver operating characteristic curve
DASS: depression, anxiety, stress, and suicidal ideation
*DSM-5*: Diagnostic and Statistical Manual of Mental Disorders, Fifth Edition
LIWC: Linguistic Inquiry and Word Count
ML: machine learning
NLP: natural language processing
SVM: support vector machine
TF-IDF: term frequency–inverse document frequency
TNB: transgender and nonbinary
XGBoost: extreme gradient boosting

## Data Availability

The preprocessed, deidentified data were not released publicly to protect transgender and nonbinary Reddit users but are freely available to qualified researchers upon request. Interested parties should contact the first author with (1) the specific ways in which they intend to use the data and (2) their safeguards for protecting the data.
