# Supplementary material for: Models of Gender Dysphoria Using Social Media Data for Use in Technology-Delivered Interventions: Machine Learning and Natural Language Processing Validation Study
Source: JMIR Form Res. 2023 Jun 16;7:e47256. doi: 10.2196/47256 (PMC10337393; doi:10.2196/47256)
Supplement: Multimedia Appendix 1 [file formative_v7i1e47256_app1.pdf]

Multimedia Appendix

This is a Multimedia Appendix to a full manuscript published in the JMIR Formative Research. For full copyright and citation information see http://dx.doi.org/10.2196/jmir.47256

Figure 1S

Top 10 Independent Variables Contributing to Misclassification of Gender Dysphoria

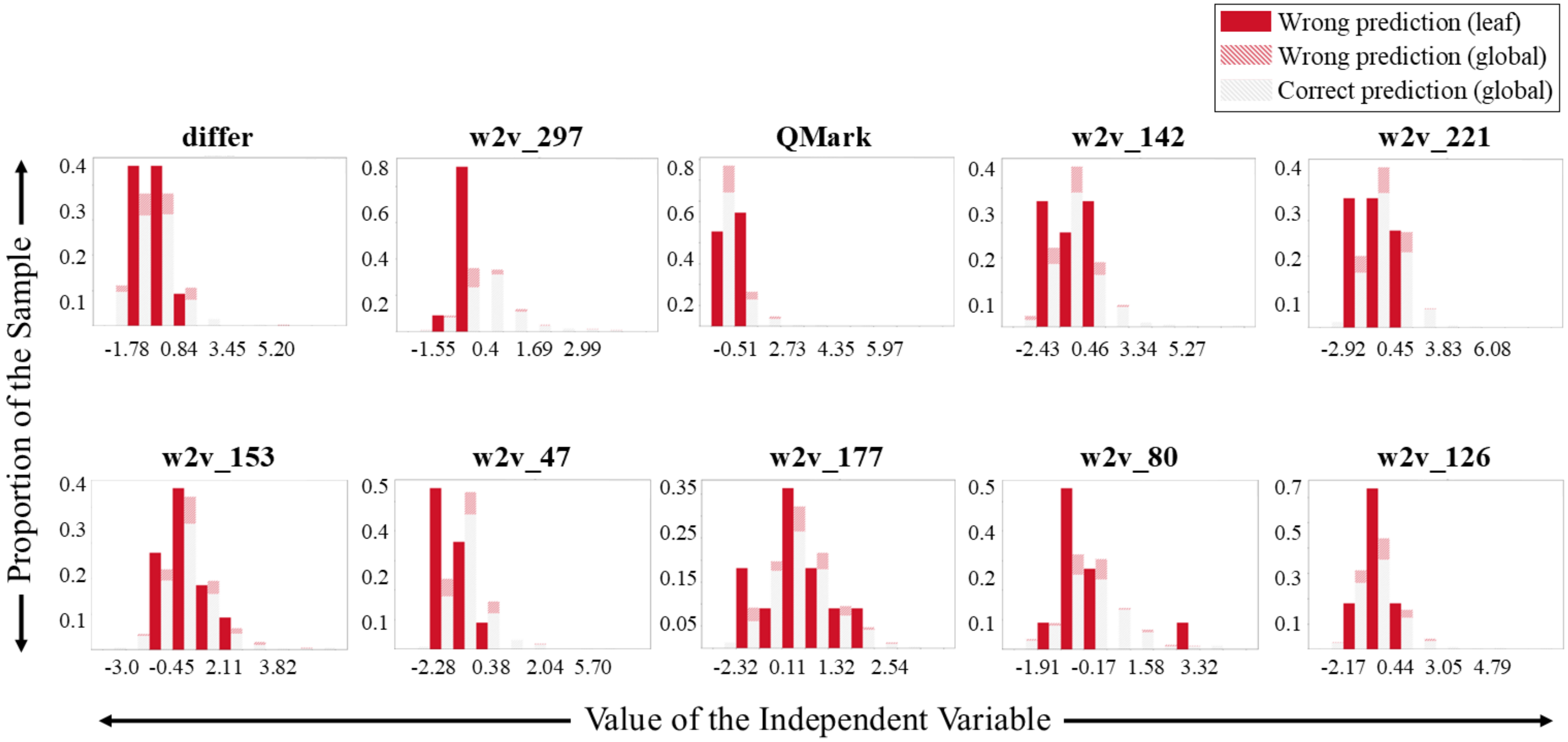

Note. Results from the error tree model [41]. All values are standardized. Wrong predictions in the leaf node (i.e., red) are compared to

wrong (i.e., red stripes) and correct (i.e., light grey stripes) predictions in the global, ground truth data set. For example, when a Reddit post is misclassified, then the value of w2v_221 (i.e., the 221st dimension of the word embedding) is most likely to be between −2.92 and 0.45; however, if the Reddit post is classified correctly, then w2v_221 is most likely to be above −2.92 and range upwards to 3.83, with the greatest proportion of correct predictions occurring just below 0.45. Independent variables in the upper left-hand corner of the figure (i.e., differ, w2v_297, etc.) are more correlated with the misclassification error than independent variables in the lower right-hand corner of the figure (i.e., w2v_80, w2v_126).

Figure 2S

XGBoost Classification Performance on the Test Data

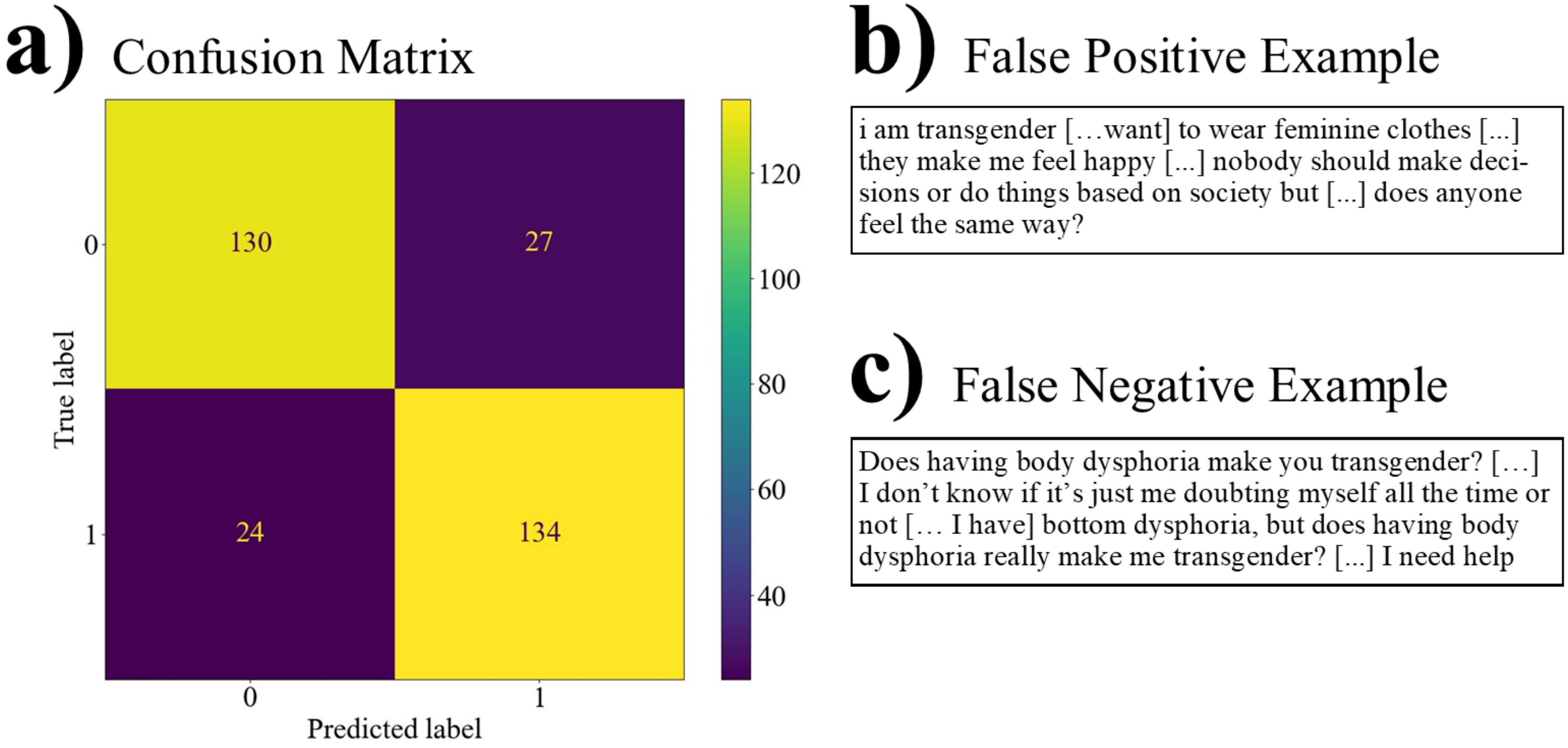

Note. In the figure, a) shows a confusion matrix of the test data, where the upper right quadrant shows the number of true negatives, the upper left quadrant shows the number of false positives, the lower right quadrant shows the number of false negatives, and the lower left quadrant shows the number of true positives; b) shows a Reddit post classified as exhibiting gender dysphoria when the qualitative coders labeled the example as not showing evidence of gender dysphoria; and c) shows a Reddit post classified as exhibiting no gender dysphoria when qualitative analysis indicated that this example shows evidence of gender dysphoria. The false positive does not indicate gender dysphoria because the Reddit user is expressing gender euphoria (i.e., a desire to wear feminine

clothes without evidence of distress) and commenting on an unjust society. The false negative actually shows evidence of dysphoria because the Reddit user is naming dysphoric areas of their body (i.e., “bottom dysphoria,” which means dysphoria related to genitalia and lower-half secondary sex characters, such as large hips). Text has been cleaned, truncated, and slightly edited to improve readability and protect the confidentiality of Reddit users.

Figure 3S

Distribution of Number of Words in Reddit Posts

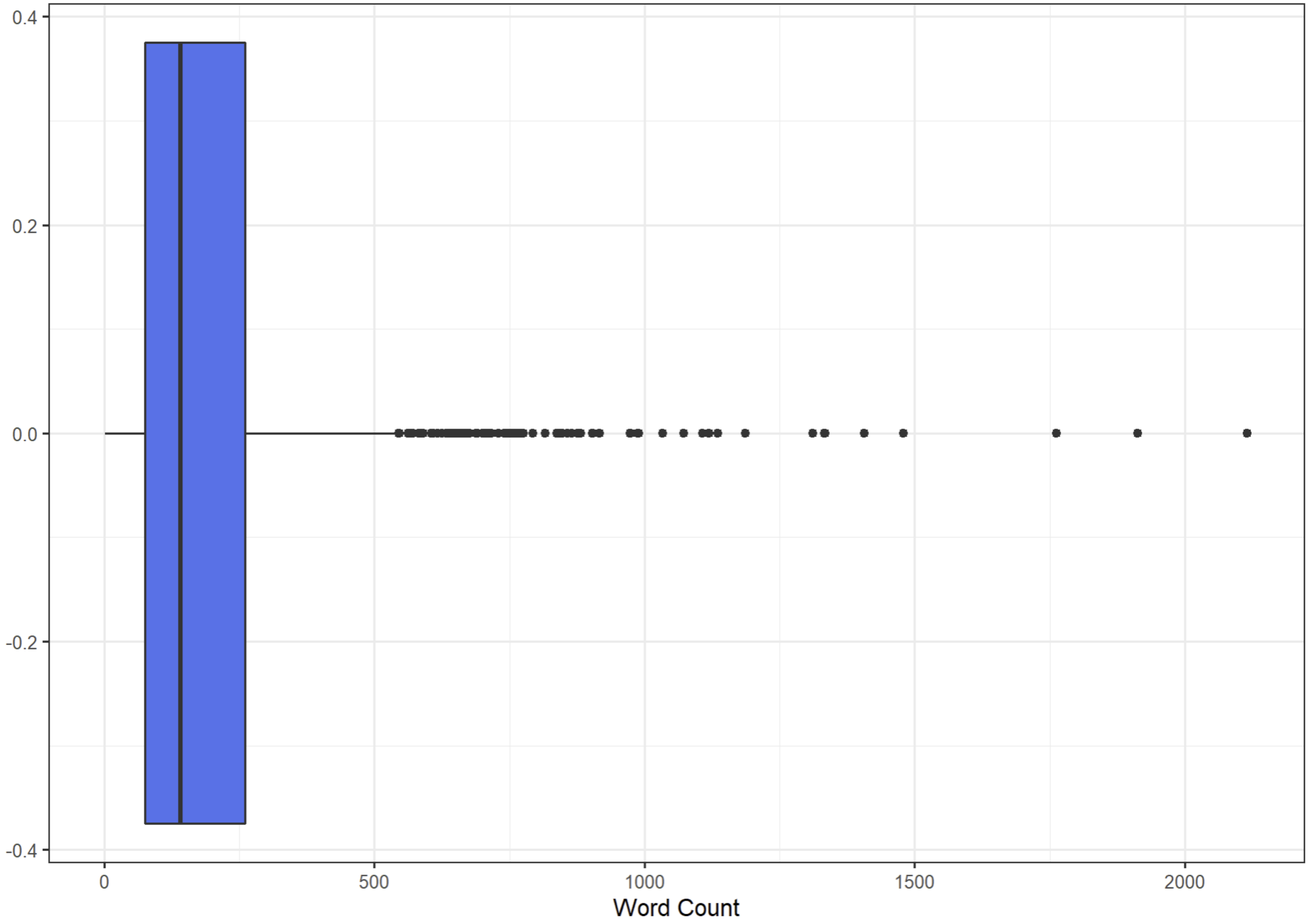

Table 1S

Gender Dysphoria Codebook

Criterion	Definition
**Manifestation of Dysphoria:** Reddit user must show evidence of at least one of the following:	
Strong desire	• Desire to remove sex characteristics related to natal sex<br>• Desire for the sex characteristics of other genders/sexes<br>• Desire to be another gender<br>• Desire to be treated as the other gender
Strong conviction	• Conviction that one has feelings typical of another gender
Perceived incongruence	• Incongruence between one's gender and one's sex characteristics (e.g., genitalia, breasts, facial hair)
Disliking identity	• Disliking how their transgender/nonbinary identity manifests in gender, mannerism, voice, body, sexual desires, and social interactions
"Feeling dysphoric"	• Naming their dysphoria directly (e.g., "I feel so dysphoric rn")<br>○ Code even if a lot of positive experiences are mentioned *if they are still working through it*<br>○ Code even if taking steps towards resolution *if still an issue*
**Negative Consequence of Dysphoria:** In addition to one of the above criteria, the Reddit user must also show evidence of:	
Distress	• Distress about their assigned gender vs. gender identity (e.g., experiencing mental health symptoms as a result of their dysphoria)
Impairment	• Impairment related to their assigned gender vs. gender identity (e.g., being unable to function, avoiding situations due to feelings of dysphoria)

Note. Codebook based on previous research [2,38].

Table 2S

Feature Selection Results of XGBoost Ranked by Feature Importance

Individual Feature Names	Feature Category	Feature Importance
clinical_keywords	clinical_keywords	35.42231
body	psycholinguistic	7.351754
w2v_235	embedding	6.480116
Tone	psycholinguistic	6.036948
w2v_159	embedding	5.453557
Clout	psycholinguistic	5.324075
i	psycholinguistic	5.29278
w2v_75	embedding	5.029416
insight	psycholinguistic	4.833448
w2v_248	embedding	4.511907
w2v_22	embedding	4.223056
w2v_2	embedding	4.065129
work	psycholinguistic	3.865027
w2v_81	embedding	3.847629
cross dress	n_grams	3.811873
w2v_208	embedding	3.710242
w2v_27	embedding	3.683938
w2v_153	embedding	3.647629
w2v_154	embedding	3.580801
w2v_125	embedding	3.475061
health	psycholinguistic	3.368988
w2v_118	embedding	3.367521
w2v_37	embedding	3.360488
dass_suicide	dass	3.326251
w2v_140	embedding	3.305332
w2v_232	embedding	3.266136
w2v_107	embedding	3.264956
verb	psycholinguistic	3.247548
w2v_223	embedding	3.246941
w2v_53	embedding	3.104581
adverb	psycholinguistic	3.076859
w2v_68	embedding	3.043096
confuse	n_grams	2.968529
Exclam	psycholinguistic	2.910899
female	psycholinguistic	2.887445
w2v_33	embedding	2.863019
w2v_72	embedding	2.86205
w2v_151	embedding	2.839965
w2v_291	embedding	2.830364
negemo	psycholinguistic	2.737072

Colon	psycholinguistic	2.707291
w2v_271	embedding	2.70518
w2v_258	embedding	2.662733
w2v_87	embedding	2.661256
w2v_189	embedding	2.660166
Authentic	psycholinguistic	2.629561
close friend	n_grams	2.621521
w2v_132	embedding	2.580297
w2v_138	embedding	2.556373
w2v_105	embedding	2.534403
w2v_117	embedding	2.520353
w2v_247	embedding	2.507159
w2v_64	embedding	2.505763
tentat	psycholinguistic	2.503419
space	psycholinguistic	2.473854
w2v_210	embedding	2.449131
w2v_167	embedding	2.425009
w2v_34	embedding	2.415523
Comma	psycholinguistic	2.415085
leisure	psycholinguistic	2.404285
w2v_214	embedding	2.366227
w2v_165	embedding	2.344533
w2v_66	embedding	2.333916
focusfuture	psycholinguistic	2.323912
w2v_181	embedding	2.307613
w2v_131	embedding	2.29921
w2v_242	embedding	2.295381
Quote	psycholinguistic	2.278764
focuspresent	psycholinguistic	2.253223
w2v_292	embedding	2.206694
w2v_60	embedding	2.187886
w2v_175	embedding	2.167437
w2v_259	embedding	2.141259
w2v_280	embedding	2.134815
affect	psycholinguistic	2.131927
AllPunc	psycholinguistic	2.130327
SemiC	psycholinguistic	2.12986
w2v_152	embedding	2.129544
w2v_264	embedding	2.11762
w2v_289	embedding	2.111741
w2v_3	embedding	2.110303
drives	psycholinguistic	2.084727
w2v_109	embedding	2.075537
w2v_285	embedding	2.074821
time	psycholinguistic	2.074678

w2v_163	embedding	2.048105
w2v_156	embedding	2.041314
w2v_287	embedding	2.031608
w2v_224	embedding	2.023989
w2v_30	embedding	2.001317
w2v_284	embedding	1.990717
w2v_88	embedding	1.988728
w2v_188	embedding	1.973696
w2v_146	embedding	1.972976
w2v_122	embedding	1.967508
w2v_6	embedding	1.96622
w2v_147	embedding	1.95913
w2v_46	embedding	1.956224
w2v_150	embedding	1.930907
shehe	psycholinguistic	1.9279
bio	psycholinguistic	1.918445
w2v_69	embedding	1.879505
w2v_198	embedding	1.872913
w2v_276	embedding	1.862973
w2v_54	embedding	1.860777
w2v_298	embedding	1.856817
w2v_160	embedding	1.851676
w2v_38	embedding	1.850132
dass_anxiety	dass	1.849875
w2v_215	embedding	1.847028
w2v_268	embedding	1.83813
w2v_169	embedding	1.831299
w2v_28	embedding	1.822702
w2v_78	embedding	1.812654
w2v_25	embedding	1.809049
death	psycholinguistic	1.793209
w2v_155	embedding	1.764504
interrog	psycholinguistic	1.760228
w2v_74	embedding	1.750057
w2v_201	embedding	1.748547
w2v_104	embedding	1.741546
w2v_205	embedding	1.739756
w2v_97	embedding	1.725223
w2v_277	embedding	1.716644
w2v_9	embedding	1.70134
w2v_18	embedding	1.698928
w2v_170	embedding	1.694242
Dash	psycholinguistic	1.691842
w2v_43	embedding	1.69041
w2v_19	embedding	1.688794

w2v_294	embedding	1.66918
sentiment_valence	sentiment_valence	1.664114
you	psycholinguistic	1.662403
w2v_251	embedding	1.648209
w2v_15	embedding	1.642578
w2v_21	embedding	1.637666
w2v_110	embedding	1.625056
w2v_250	embedding	1.619029
w2v_70	embedding	1.610045
Analytic	psycholinguistic	1.608044
w2v_168	embedding	1.596145
w2v_212	embedding	1.593892
negate	psycholinguistic	1.576869
swear	psycholinguistic	1.570817
w2v_100	embedding	1.563693
w2v_55	embedding	1.56368
w2v_164	embedding	1.557301
affiliation	psycholinguistic	1.537246
w2v_244	embedding	1.536771
w2v_86	embedding	1.533373
function_liwc	psycholinguistic	1.529226
w2v_162	embedding	1.517123
friend	psycholinguistic	1.514672
we	psycholinguistic	1.50486
w2v_73	embedding	1.502176
w2v_270	embedding	1.500945
w2v_200	embedding	1.496209
w2v_108	embedding	1.494489
w2v_166	embedding	1.49249
w2v_58	embedding	1.488058
w2v_229	embedding	1.477272
w2v_192	embedding	1.469408
w2v_26	embedding	1.464232
w2v_84	embedding	1.450071
w2v_52	embedding	1.444341
differ	psycholinguistic	1.444107
w2v_11	embedding	1.431513
w2v_293	embedding	1.419867
w2v_112	embedding	1.418813
WPS	psycholinguistic	1.403735
w2v_176	embedding	1.401908
w2v_36	embedding	1.400462
w2v_137	embedding	1.398359
w2v_141	embedding	1.361732
w2v_243	embedding	1.355055

w2v_172	embedding	1.348801
w2v_231	embedding	1.348002
w2v_29	embedding	1.336319
w2v_187	embedding	1.333343
w2v_196	embedding	1.324101
w2v_17	embedding	1.323398
w2v_89	embedding	1.323286
w2v_121	embedding	1.318395
w2v_281	embedding	1.317865
assent	psycholinguistic	1.308626
social	psycholinguistic	1.307979
w2v_177	embedding	1.306319
w2v_184	embedding	1.305332
w2v_62	embedding	1.301102
w2v_0	embedding	1.298881
w2v_178	embedding	1.297483
w2v_96	embedding	1.294888
w2v_23	embedding	1.294206
w2v_226	embedding	1.290789
w2v_237	embedding	1.287678
risk	psycholinguistic	1.281763
w2v_220	embedding	1.276322
w2v_158	embedding	1.267275
w2v_263	embedding	1.266588
w2v_253	embedding	1.266491
w2v_123	embedding	1.26405
w2v_278	embedding	1.262973
w2v_61	embedding	1.25278
w2v_94	embedding	1.249112
w2v_93	embedding	1.241144
w2v_82	embedding	1.237042
w2v_142	embedding	1.230359
w2v_92	embedding	1.222119
Apostro	psycholinguistic	1.221618
WC	psycholinguistic	1.214979
w2v_115	embedding	1.212863
ears	n_grams	1.207941
w2v_266	embedding	1.207074
w2v_282	embedding	1.206909
w2v_148	embedding	1.200713
compare	psycholinguistic	1.195421
power	psycholinguistic	1.179621
w2v_222	embedding	1.17116
w2v_221	embedding	1.165515
w2v_191	embedding	1.163608

w2v_98	embedding	1.163264
w2v_246	embedding	1.157841
w2v_57	embedding	1.152349
w2v_228	embedding	1.140721
w2v_12	embedding	1.137474
w2v_76	embedding	1.136045
w2v_217	embedding	1.134791
w2v_51	embedding	1.133205
w2v_161	embedding	1.130748
w2v_50	embedding	1.129256
Parenth	psycholinguistic	1.125925
w2v_13	embedding	1.123646
w2v_179	embedding	1.120524
w2v_4	embedding	1.117184
w2v_83	embedding	1.10091
focuspast	psycholinguistic	1.09891
informal	psycholinguistic	1.096981
w2v_47	embedding	1.090972
cogproc	psycholinguistic	1.089305
w2v_274	embedding	1.087191
w2v_129	embedding	1.080893
certain	psycholinguistic	1.080626
w2v_255	embedding	1.074022
w2v_171	embedding	1.073331
w2v_145	embedding	1.067307
feel	psycholinguistic	1.051698
home	psycholinguistic	1.028073
w2v_40	embedding	1.027758
w2v_206	embedding	1.026274
w2v_218	embedding	1.023692
w2v_204	embedding	1.023523
w2v_262	embedding	1.02259
w2v_236	embedding	1.016844
percept	psycholinguistic	1.015237
w2v_42	embedding	1.01089
w2v_234	embedding	1.009849
relativ	psycholinguistic	1.007981
w2v_113	embedding	0.989964
cause	psycholinguistic	0.989745
w2v_267	embedding	0.986074
w2v_80	embedding	0.983608
w2v_85	embedding	0.98359
w2v_56	embedding	0.982348
Period	psycholinguistic	0.977454
w2v_133	embedding	0.973015

w2v_8	embedding	0.965861
w2v_193	embedding	0.94761
w2v_24	embedding	0.947133
family	psycholinguistic	0.945385
w2v_245	embedding	0.940113
w2v_269	embedding	0.932258
w2v_216	embedding	0.931193
w2v_59	embedding	0.926741
w2v_239	embedding	0.921545
w2v_31	embedding	0.921197
w2v_256	embedding	0.920574
w2v_1	embedding	0.917462
w2v_106	embedding	0.916919
w2v_288	embedding	0.916478
w2v_213	embedding	0.911775
discrep	psycholinguistic	0.903847
hear	psycholinguistic	0.902591
OtherP	psycholinguistic	0.898403
quant	psycholinguistic	0.897571
motion	psycholinguistic	0.895011
w2v_297	embedding	0.892278
w2v_252	embedding	0.890616
w2v_275	embedding	0.883192
w2v_5	embedding	0.88042
w2v_194	embedding	0.877309
posemo	psycholinguistic	0.873679
Sixltr	psycholinguistic	0.871848
w2v_265	embedding	0.870062
w2v_41	embedding	0.866054
cam	n_grams	0.858752
w2v_299	embedding	0.857276
w2v_10	embedding	0.840551
QMark	psycholinguistic	0.839139
w2v_77	embedding	0.831429
w2v_257	embedding	0.825859
w2v_272	embedding	0.821956
article	psycholinguistic	0.817601
w2v_124	embedding	0.812012
adj	psycholinguistic	0.806192
w2v_44	embedding	0.805723
w2v_99	embedding	0.800227
w2v_67	embedding	0.792921
anx	psycholinguistic	0.788078
auxverb	psycholinguistic	0.778735
w2v_202	embedding	0.777765

nonflu	psycholinguistic	0.772544
w2v_63	embedding	0.772022
w2v_130	embedding	0.763425
w2v_136	embedding	0.762608
w2v_185	embedding	0.759135
w2v_39	embedding	0.757782
w2v_295	embedding	0.753686
w2v_49	embedding	0.739417
w2v_203	embedding	0.73545
w2v_16	embedding	0.731376
w2v_183	embedding	0.72609
w2v_14	embedding	0.725563
w2v_249	embedding	0.724093
number	psycholinguistic	0.713746
filler	psycholinguistic	0.711098
w2v_209	embedding	0.709318
sad	psycholinguistic	0.705832
ingest	psycholinguistic	0.7043
w2v_45	embedding	0.698968
w2v_173	embedding	0.695266
w2v_290	embedding	0.694124
w2v_261	embedding	0.692756
sexual	psycholinguistic	0.692233
w2v_157	embedding	0.69139
reward	psycholinguistic	0.690784
w2v_32	embedding	0.679287
w2v_95	embedding	0.653442
w2v_207	embedding	0.64468
w2v_227	embedding	0.640155
w2v_225	embedding	0.638978
w2v_35	embedding	0.626316
Dic	psycholinguistic	0.624229
w2v_7	embedding	0.623847
w2v_103	embedding	0.62259
w2v_197	embedding	0.621924
ipron	psycholinguistic	0.617778
w2v_134	embedding	0.616879
w2v_91	embedding	0.616136
w2v_260	embedding	0.614236
w2v_241	embedding	0.612385
w2v_149	embedding	0.605911
w2v_144	embedding	0.604439
w2v_111	embedding	0.60001
w2v_190	embedding	0.578901
pronoun	psycholinguistic	0.574935

w2v_120	embedding	0.574274
money	psycholinguistic	0.570538
w2v_65	embedding	0.559452
w2v_101	embedding	0.549841
w2v_135	embedding	0.54556
w2v_230	embedding	0.540174
w2v_143	embedding	0.538158
w2v_79	embedding	0.533182
w2v_219	embedding	0.525499
w2v_195	embedding	0.520341
w2v_48	embedding	0.506503
w2v_238	embedding	0.503626
w2v_273	embedding	0.497744
w2v_116	embedding	0.496389
prep	psycholinguistic	0.492794
male	psycholinguistic	0.479721
conj	psycholinguistic	0.436974
w2v_126	embedding	0.434827
w2v_199	embedding	0.429015
w2v_240	embedding	0.42739
w2v_296	embedding	0.417654
w2v_283	embedding	0.416865
w2v_211	embedding	0.414101
w2v_139	embedding	0.408748
w2v_119	embedding	0.408113
w2v_20	embedding	0.404642
w2v_127	embedding	0.387941
w2v_186	embedding	0.37726
w2v_286	embedding	0.346013
they	psycholinguistic	0.332965
w2v_182	embedding	0.315825
ppron	psycholinguistic	0.310122
netspeak	psycholinguistic	0.301971
w2v_90	embedding	0.287345
w2v_102	embedding	0.265739
w2v_279	embedding	0.260967
w2v_254	embedding	0.211694
w2v_174	embedding	0.187861
see	psycholinguistic	0.137872
w2v_71	embedding	0.090867
achieve	psycholinguistic	0.086899

Note. Feature importance calculated as information gain.

Table 3S

Performance Metric from Training the DASS Classifiers

**Performance Metric**	**Depression SVM**	**Anxiety SVM**	**Stress SVM**	**Suicidal Ideation SVM**
Training data				
Accuracy	0.902	0.937	0.931	0.902
Precision	0.954	0.977	0.996	0.956
Recall	0.845	0.895	0.866	0.842
F1	0.897	0.934	0.926	0.896
Testing data				
Accuracy	0.904	0.938	0.926	0.905
Precision	0.954	0.980	0.995	0.959
Recall	0.848	0.894	0.857	0.847
F1	0.898	0.935	0.921	0.899

Note. SVM = support vector machine.
